# Immunohistochemical expression of interleukin-17 and hormonal receptors in benign and malignant breast lesions

**DOI:** 10.1186/s13104-020-05146-7

**Published:** 2020-06-23

**Authors:** Eman Taha Ali, Mai Abdulrahman Mohammed Masri, Emmanuel Edwar Siddig, Ayman Ahmed, Mohamed S. Muneer, Nouh Saad Mohamed, Ali Mahmoud Mohammed Edris

**Affiliations:** 1 Department of Histopathology and Cytology, Faculty of Medical Laboratory Sciences, University of Khartoum, Khartoum, Sudan; 2 Department of Histopathology and Cytology, Faculty of Medical Laboratory Sciences, National University, Khartoum, Sudan; 3 Molecular Biology Department, Faculty of Zoology, University of Khartoum, Khartoum, Sudan; 4 Mycetoma Research Center, University of Khartoum, Khartoum, Sudan; 5 School of Medicine, Nile College, Khartoum, Sudan; 6 Department of Histopathology and Cytology, Alfarrabi College for Science and Technology, Khartoum, Sudan; 7 Institute of Endemic Diseases, University of Khartoum, Khartoum, Sudan; 8 Department of Neurology, Mayo Clinic, Jacksonville, FL USA; 9 Department of Radiology, Mayo Clinic, Jacksonville, FL USA; 10 Department of Internal Medicine, Faculty of Medicine, University of Khartoum, Khartoum, Sudan; 11 Department of Parasitology and Medical Entomology, Alfarrabi College for Science and Technology, Khartoum, Sudan; 12 Department of Parasitology and Medical Entomology, Faculty of Medical Laboratory Sciences, Nile University, Khartoum, Sudan; 13 Department of Parasitology, Faculty of Medicine, Sinnar University, Sinnar, Sudan; 14 Department of Histopathology and Cytology, Faculty of Applied Medical Sciences, University of Bisha, Bisha, Kingdom of Saudi Arabia

**Keywords:** Interleukin-17, Immunohistochemistry, Breast lesions, Breast cancer

## Abstract

**Objectives:**

IL17 is a critical pro-inflammatory cytokine that is involved in inflammation, multidrug resistance and growth persistence pathways in cancer. This study is aiming at studying the expression of IL17 and hormonal receptors expression in benign and malignant breast lesions using immunohistochemical staining methods.

**Results:**

A total of 137 cases of breast lesions were studied, 97 (70.8%) were malignant and 40 (29.2%) were benign cases. Age range for malignant and benign cases were between 26 and 80 years [mean age 50 ± 2 years], and 20 to 70 years [mean age 41 ± 4 years], respectively, Odds ratio = 2.3 [1.78–1.99, 95% CI]. The majority of the histopathological diagnosis of the benign and malignant lesions were 21 (15.3%) fibro-adenomas and 87 (63.5%) invasive ductal carcinoma, respectively. Expression of IL17 and age were insignificantly negatively correlated for both groups; benign cases [r = − 0.054, P value 0.742] and malignant cases [r = − 0.080, P value 0.444]. IL17 expression was showing insignificant association with age group, P value 0.065. IL17 expression showed a statistical significance based on the different histopathological diagnosis, P value 0.035. Expression levels of estrogen, progesterone, and human epidermal receptors were showing insignificant difference among IL17 expression categories, P values 0.678, 0.623, and 0.361, respectively.

## Introduction

Breast cancer (BC) is one of the global prevalent malignancies among females [1]. According to 2019 cancer statistics, the incidence of BC is surprisingly increasing compared to earlier reports in 2018. Not surprisingly, the rate of mortality is very high in the African developing countries owing to socioeconomic factors [2]. Importantly, the majority of women in these countries were diagnosed at late stages of the disease that ominously, has been metastasized [2]. In a previous study, among 120 patients diagnosed with benign lesion and were followed up for almost 20 years, 34% of all palpable lesions were found to be malignant tumors followed by fibro-adenoma (28%), fibrocystic diseases (11%), inflammatory changes (11%), and other phylloid tumors and lactation changes (6%) [3]. According to the WHO there are several features in present in the benign lesions that are well known as pre-cancerous signs [4].

Metastasis and drug resistance constitute important problems in BC management and survival as well [5]. Lately, studies investigated the role of cytokines expression among both benign and malignant cancer patients, their finding was quite unique to further investigate the role of cytokines expression to understand the invasion mechanism, then as diagnostic and prognostic markers for cancer development [6, 7].

Recently, cytokines and other ligands of immune system have been reported as instrumental factors in tumor microenvironment, and many of them are considered as prognostic and therapeutic targets in cancer [8, 9]. Basically, *Interleukin*-*17* (*IL17*) is one of these important mediators which have strong potential mechanism to induce inflammation by enhancing neutrophil migration, angiogenesis, and matrix metalloproteinase production [10–12]. Furthermore, *IL17* can induce other important signaling pathways in tumor cell itself such as *Kras* and epithelial-mesenchymal transition which were reported as imperative pathways in cancer growth and metastasis [12–14]. *IL17* was also involved by triggering several pathways in tumor that lead to growth persistence against cancer therapy [15].

Status of steroid and growth factor receptors expression such as Estrogen Receptor (ER), Progesterone Receptor (PR), and Hunan Epidermal Receptor-2 (HER2) play a key role in term of diagnosis and treatment of BC [16–18], since *IL17* is directly related to production of steroid hormones and growth factors [9, 19, 20]. Consequently, the production of *IL17* in tumor microenvironment suggested to play other significant clinical roles in BC development [9, 19, 20]. Previous facts indicate a crucial role of *IL17* in cancer initiation, prognosis and treatment. Therefore, giving great interest to study its expression in benign and malignant breast lesions and with this background our current study is aiming at studying the expression of *IL17* and hormonal expression in benign and malignant breast lesions using Immunohistochemical staining Methods (IHC).

## Main text

### Materials and methods

#### Study design and sample collection

A descriptive cross-sectional hospital-based study conducted in National Central Khartoum Lab between 2017 and 2018. Simple random sampling method has been done; to collect Archival paraffin blocks from 127 female patients diagnosed with any type of breast lesion. Patients’ age and diagnosis were obtained from hospital’s record. Cancer grade and immunohistochemical expression of ER, PR, and HER2 for malignant cases were also obtained from the records which are done according to automated Ventana Benchmark XT system. ER and PR were graded using Allred scoring system [21]. HER2 was evaluated based on the membranous staining intensity according to Wolff et al., 2007 [22].

#### Paraffin sections preparation and Immunohistochemical Technique

One paraffin sections of 3–5 micrometers thickness was cut for IHC staining using rotary microtome. Immunohistochemical expression of *IL17* was detected according to manufacturer instructions using the commercially available kit Mouse and Rabbit specific HRP/DAB Detection kit (ab64264, Abcam, Cambridge, UK). Briefly, tissue sections were de-waxed by xylene and rehydrated through gradients ethanol into water. For antigen retrieval, sections were heated in Citrate buffer (pH 6.0) for 20 min at 95 ^°^C and quenched for endogenous peroxidase activity using 3% H_2_O_2_ in methyl alcohol. Sections were then washed in phosphate buffer saline (PBS) and the nonspecific binding of protein and antibody was blocked using protein block, then protein block was blotted off. Sections were incubated overnight at 4 °C with primary antibody against *IL17* (ab136668) at 1:100 dilution, after adjustment of the dilution and incubation period by using tonsil as a positive control for this antigen. After that, sections were washed with PBS then incubated with biotinylated secondary antibody. After several proper washes with washing buffer, the color was developed using HRP/DAP kit. Sections were lightly counterstained with hematoxylin and blued by running tap water, followed by dehydration and mounting in DPX.

#### Immunohistochemical scoring system

The cytoplasmic staining scoring for *IL17* in all tissue sections was performed semi-quantitatively based on the overall intensity of staining on the cytoplasm by two different pathologists into no expression, mild expression, moderate expression, and strong expression, according to the overall percentage of stained cells; no cells express the *IL17*, 10–30% of cells express *IL17*, 40–60% of cells express *IL17*, 60–80% of cells express *IL17*, and > 80% of the cells express *IL17*, respectively.

### Statistical analysis

Data were analysed using the Statistical Package for Social Science (SPSS, v16). Chi Square test was used to analyze different categories a *P* value of < 0.05 was considered statistically significant. Pearson correlation of *IL17* expression in regards to age of cases and hormonal receptors expression besides Odds ratios were also calculated.

### Results

#### Patients demographics and sample categorization

The present study included 137 cases of breast lesions. Their age ranged between 20 and 80 years [mean age of 47 ± 2 years]. Notably, the age group 41 to 60 years constituted the majority of studied population; 85 (62.0%). When categorizing the breast lesions-based malignancy, a total of 97 (70.8%) were malignant and 40 (29.2%) were benign. The malignant cases their age ranged between 26 and 80 years [mean of 50 ± 2 years], whereas, the benign cases their age ranged between 20 and 70 years [mean age of 41 ± 4 years], Odds ratio = 2.3 [1.78–1.99, 95% CI].

The benign cases based on the histopathological diagnosis included; fibro-adenomas (FA) which constituted the vast majority of the benign lesions 21 (15.3%), followed by duct ectasia (DE) 11 (8.0%), fibrocystic change (FC) 4 (2.9%), lipoma 2 (1.5%), and benign phyllodes tumor (PTS) 2 (1.5%). While the malignant cases included 87 (63.5%) cases reported as invasive ductal carcinoma (IDC), 7 (5.1%) cases were invasive lobular carcinoma (ILC) and 3 (2.2%) cases were intraductal carcinoma in situ (IDCS). The type of breast lesion was statistically significant with age group, P value 0.003 as well, classification based on the histopathological examination was showing significant association when grouped according to age group, P value 0.000.

#### *IL17* immunohistochemical expression

The expression of *IL17* and age of cases were insignificantly negatively correlated for both groups; benign cases [r = − 0.054, P value 0.742] and malignant cases [r = − 0.080, P value 0.444]. The levels of *IL17* expression among cases aged between 41 and 60 years, more than 60 years, and between 21 and 40 years were 29.4% strong, 47.1% moderate, and 16.7% mild, respectively. *IL17* expression was showing insignificant association when categorized based on age groups, P value 0.065.

**Fig. 1:**
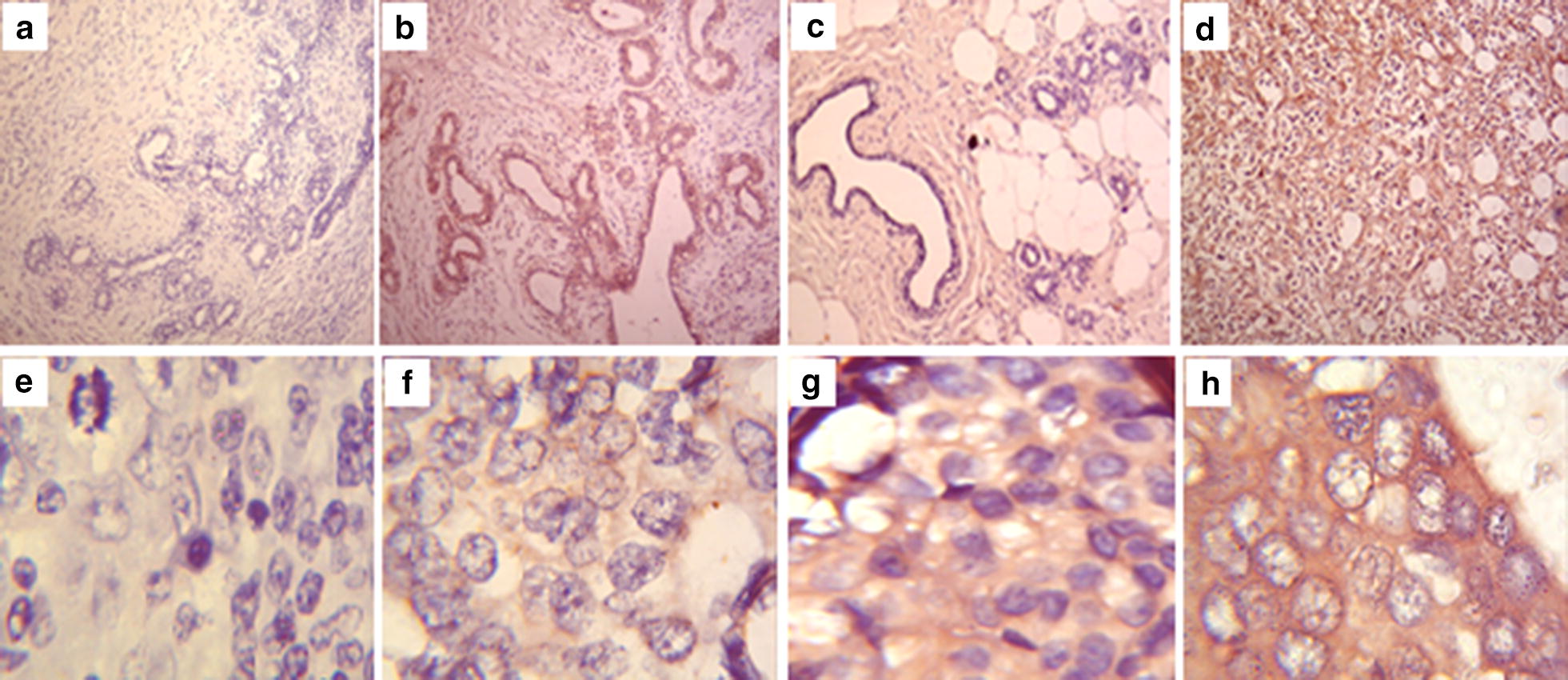
Immunohistochemical expression of IL17 in malignant and benign tissues. Sections stained with Mayer’s hematoxylin-DAP peroxidase. Microscopic magnification ×10; **a** negative benign FA section, **b** positive benign FA section, **c** negative malignant IDC section, and **d** positive malignant IDC section. Microscopic magnification ×40; **e** negative malignant IDC section, **f** mild positive malignant IDC section, **g** moderately positive malignant IDC section, **h** strongly positive malignant IDC section

*IL17* expressed in 63/97 (64.9%) of the malignant cases and 17 (42.5%) of the benign cases. Mild expression of *IL17* was noted among 22 (22.7%) of the malignant cases, while moderate expression was recorded among 10 (25.0%) of the benign cases and 15 (15.5%) of the malignant cases. Whereas benign and malignant cases recorded to show strong *IL17* expression were 7 (17.5%) and 26 (26.8%), respectively (Fig. 1). A statistical significance was noted for the different expression categories among the benign and malignant cases, P value 0.002.

A strong expression of *IL17* in malignant tissue sections was 28.7% among DC cases and 33.3% in IDC cases. For ILC cases, mild and moderate expression of *IL17* was 28.6% for each. However, among the benign cases, DE and FA were mostly showing moderate expression; 36.4% and 28.6%, respectively. While PTS and FC were showing strong expression; 100% and 50.0%, correspondingly. Benign cases which diagnosed histopathologically as lipoma did not showed any expression of *IL17*.

*IL17* expression showed a statistical significance based on the different histopathological diagnosis, P value 0.035. With respect to hormonal receptors expression, the expression levels of ER, PR, and HER2 were showing insignificant difference among the different *IL17* expression categories, P values 0.678, 0.623, and 0.361, respectively. The results of *IL17* expression based on age groups, tumor type, histopathological diagnosis and hormonal receptors were illustrated in Table 1.

**Table 1:** Relationship of different variables in study population with IL17 expression

	IL17 expression				Total (n = 137)	P value
	No expression	Mild	Moderate	Strong		
Age group						
Not more than 20	1 (100%)	0 (0.0%)	0 (0.0%)	0 (0.0%)	1 (0.7%)	0.065
21 to 40 years	13 (43.3%)	5 (16.7%)	7 (23.3%)	5 (16.7%)	30 (21.9%)	
41 to 60 years	38 (44.7%)	13 (15.3%)	9 (10.6%)	25 (29.4%)	85 (62.0%)	
Above 60 years	5 (29.4%)	2 (11.8%)	8 (47.1%)	2 (11.8%)	17 (12.4%)	
Tumor type						
Benign	23 (57.5%)	0 (0.0%)	10 (25.0%)	7 (17.5%)	40 (29.2%)	0.002
Malignant	34 (34.1%)	22 (22.7%)	15 (15.5%)	26 (26.8%)	97 (70.8%)	
Diagnosis of breast lesion						
IDC	30 (34.5%)	19 (21.8%)	13 (14.9%)	25 (28.7%)	87 (63.5%)	0.035
DE	7 (63.6%)	0 (0.0%)	4 (36.4%)	0 (0.0%)	11 (8.0%)	
FA	12 (57.1%)	0 (0.0%)	6 (28.6%)	3 (14.3%)	21 (15.3%)	
FC	2 (50.0%)	0 (0.0%)	0 (0.0%)	2 (50.0%)	4 (2.9%)	
IDCS	2 (66.7%)	1 (33.3%)	0 (0.0%)	0 (0.0%)	3 (2.2%)	
ILC	2 (28.6%)	2 (28.6%)	2 (28.6%)	1 (14.3%)	7 (5.1%)	
Lipoma	2 (100%)	0 (0.0%)	0 (0.0%)	0 (0.0%)	2 (1.5%)	
PTS	0 (0.0%)	0 (0.0%)	0 (0.0%)	2 (100%)	2 (1.5%)	
ER						
Mild	10 (37.0%)	7 (25.9%)	3 (11.1%)	7 (25.9%)	27 (19.7%)	0.678
Moderate	3 (21.4%)	4 (28.6%)	4 (28.6%)	3 (21.4%)	14 (10.2%)	
Strong	5 (29.4%)	5 (29.4%)	1 (5.9%)	6 (35.3%)	17 (12.4%)	
No expression	16 (41.0%)	6 (15.4%)	7 (17.9%)	10 (25.6%)	39 (28.5%)	
PR						
Mild	9 (37.5%)	6 (25.0%)	3 (12.5%)	6 (25.0%)	24 (17.5%)	0.623
Moderate	3 (30.0%)	3 (30.0%)	1 (10.0%)	3 (30.0%)	10 (7.3%)	
Strong	1 (8.3%)	3 (25.0%)	2 (16.7%)	6 (50.0%)	12 (8.8%)	
No expression	21 (41.2%)	10 (19.6%)	9 (17.6%)	11 (21.6%)	51 (37.2%)	
HER2						
Mild	7 (35.0%)	7 (35.0%)	0 (0.0%)	6 (30.0%)	20 (14.6%)	0.361
Moderate	7 (46.7%)	2 (13.3%)	3 (20.0%)	3 (20.0%)	15 (10.9%)	
Strong	8 (33.3%)	3 (12.5%)	4 (16.7%)	9 (37.5%)	24 (17.5%)	
No expression	12 (31.6%)	10 (26.3%)	8 (21.1%)	8 (21.1%)	38 (27.7%)	

*ER* estrogen receptor, *PR* progesterone receptor, *HER2* Human epidermal receptor-2

Concerning the correlation of ER, PR, and HER2 expression and *IL17* expression in the breast lesions, no statistically significance correlation was shown for these receptors and *IL17* expression (Table 2).

**Table 2:** The correlation of estrogen receptor, progesterone receptor, and Human epidermal receptor-2 expression with IL17 expression in the breast lesions

	Pearson’s *r*	P value	95% CI [Lower bound–upper bound]
Estrogen receptor	− 0.001	0.993	2.70 [1.44–3.96]
Progesterone receptor	− 0.024	0.813	2.93 [1.65–4.21]
Human epidermal receptor-2	0.043	0.679	2.83 [1.66–3.98]

### Discussion

Pro-inflammatory cytokines play a key role in cancer microenvironment and were associated with bad prognosis in breast cancer [7–9]. The current study has described the expression of *IL17* in different subsets of benign and malignant lesions of the breast. Notably, malignant cases had strong positive expression in both number of cells and intensity of the reaction when compared with the benign ones. These findings were consistent with previous exhaustive studies pointing that *IL17* is more associated with malignant breast lesions [23–25]. The variation in the staining patterns between IDC and IDCS found in this study can be useful in differentiation between the two BC classes. IDC can show moderate and strong expression of *IL17* compared to IDCS. These patterns variation might be attributed to the high numbers of infiltrating *IL17*-producing cells in IDC [9]. Similar results were also stated in malignant and benign lesions in the thyroid and salivary gland cancers [26, 27].

Benign lesions in this study were found to produce considerable levels of *IL17* in epithelial cells of normal ducts and lobules; the highest production was detected in PTS and FA. Nonetheless, the data regarding cytokines expression in benign tumors is limited and strongly focused on FA subset of benign tumor [28]. Thus, to our knowledge, this study is among the first to address *IL17* among other breast lesions. Positive expression was also obtained in DE and FC in which expression was restricted to the inflammatory cells and stroma. In DE, the large numbers of macrophages foamy cells expressing *IL17* might be attributed, mainly, to the inflammatory reactions associated with this condition [29].

The majority of studies demonstrated in situ expression of *IL17* in breast cancer along with T lymphocyte and innate immune cells being the main expression site for this cytokine in other types of cancers [30–32]. Recently *IL17* was shown to be expressed by other cells like plasma cells and paneth cells in gut [33, 34]. In the current study the main site of *IL17* expression was malignant cells themselves in addition to tumor stroma. This finding is in accordance with Li et al. [35] and Al-Samadi et al. [36] who found high prevalence of breast cancer and colon cancer cells expressing *IL17*, correspondingly.

Regarding the association between *IL17* expression and hormonal expression (ER, PR and HER2) among malignant cases, the obtained results disclose no significant association between *IL17* expression in breast tissue and the previously mentioned receptors (*P* values > 0.05). Conversely to our study, *IL17* was more associated with ER- and triple negative BC [9, 15]. However, in concordance to our study Chavey et al. [20] found that *IL17* is one of the cytokines that are not related with steroid receptors expression. Furthermore, Slattery et al. [37] found that the relationship between cytokines and steroid receptors expression is affected by particular variants of cytokines with no association between *IL17* variants and these receptors expression in the breast. Therefore, these results indicate that *IL17* role in breast cancer might be independent of hormonal receptors expression.

### Conclusion

*IL17* is expressed in a variety of cell types including benign and malignant cells; mostly tumor cells and tumor associated stromal cells. Malignant cells significantly express higher levels of *IL17* compared to benign tumor cells. *IL17* expression in BC might be independent of hormonal receptors expression.

## Limitations

- This study evaluated the expression of *IL17* in a small cohort of breast lesions, including benign and malignant lesions without providing dual staining to specify cells types.

## Data Availability

The datasets used and/or analyzed during the current study are available from the corresponding author on reasonable request.

## Abbreviations

BC: Breast cancer
DE: Duct ectasia
ER: Estrogen receptor
FA: Fibro-adenomas
FC: Fibrocystic change
HER2: Human epidermal receptor-2
IDC: Invasive ductal carcinoma
IDCS: Intraductal carcinoma in situ
*IL17*: *Interleukin*-*17*
ILC: Invasive lobular carcinoma
PBS: Phosphate buffer saline
PR: Progesterone receptor
PTS: Benign phyllodes tumor
